# Adaptive Dynamic Therapy and Survivorship for Operable Pancreatic Cancer

**DOI:** 10.1001/jamanetworkopen.2022.18355

**Published:** 2022-06-23

**Authors:** Samer AlMasri, Mazen Zenati, Abdulrahman Hammad, Ibrahim Nassour, Hao Liu, Melissa E. Hogg, Herbert J. Zeh, Brian Boone, Nathan Bahary, Aatur D. Singhi, Kenneth K. Lee, Alessandro Paniccia, Amer H. Zureikat

**Affiliations:** 1 Department of Surgery, University of Pittsburgh, Pittsburgh, Pennsylvania; 2 Department of Surgery, Epidemiology, Clinical and Translational Science, University of Pittsburgh, Pittsburgh, Pennsylvania; 3 Department of Surgery, University of Florida, Gainesville; 4 Department of Surgery, NorthShore Hospital System, Chicago, Illinois; 5 Department of Surgery, University of Texas Southwestern, Dallas; 6 Department of Surgery, West Virginia University, Morgantown; 7 Department of Internal Medicine, Allegheny Health Network, Pittsburgh, Pennsylvania; 8 Department of Pathology, University of Pittsburgh, Pittsburgh, Pennsylvania

## Abstract

**Importance:**

Neoadjuvant therapy is increasingly used in localized pancreatic carcinoma, and survival is correlated with carbohydrate antigen 19-9 (CA19-9) levels and histopathologic response following neoadjuvant therapy. With several regimens now available, the choice of chemotherapy could be best dictated by response to neoadjuvant therapy (as measured by CA19-9 levels and/or pathologic response), a strategy defined herein as adaptive dynamic therapy.

**Objective:**

To evaluate the association of adaptive dynamic therapy with oncologic outcomes in patients with surgically resected pancreatic cancer.

**Design, Setting, and Participants:**

This retrospective cohort study included patients with localized pancreatic cancer who were treated with either gemcitabine/nab-paclitaxel or fluorouracil, leucovorin, irinotecan, and oxaliplatin (FOLFIRINOX) preoperatively between 2010 and 2019 at a high-volume tertiary care academic center. Participants were identified from a prospectively maintained database and had a median follow-up of 49 months. Data were analyzed from October 17 to November 24, 2020.

**Exposures:**

The adaptive dynamic therapy group included 219 patients who remained on or switched to an alternative regimen as dictated by CA19-9 response and for whom the adjuvant regimen was selected based on CA19-9 and/or pathologic response. The nonadaptive dynamic therapy group included 103 patients who had their chemotherapeutic regimen selected independent of CA19-9 and/or tumoral response.

**Main Outcomes and Measures:**

Prognostic implications of dynamic perioperative therapy assessed through Kaplan-Meier analysis, Cox regression, and inverse probability weighted estimators.

**Results:**

A total of 322 consecutive patients (mean [SD] age, 65.1 [9] years; 162 [50%] women) were identified. The adaptive dynamic therapy group, compared with the nonadaptive dynamic therapy group, had a more pronounced median (IQR) decrease in CA19-9 levels (−80% [−92% to −56%] vs −45% [−81% to −13%]; *P* < .001), higher incidence of complete or near-complete tumoral response (25 [12%] vs 2 [2%]; *P* = .007), and lower median (IQR) number of lymph node metastasis (1 [0 to 4] vs 2 [0 to 4]; *P* = .046). Overall survival was significantly improved in the dynamic group compared with the nondynamic group (38.7 months [95% CI, 34.0 to 46.7 months] vs 26.5 months [95% CI, 23.5 to 32.9 months]; *P* = .03), and on adjusted analysis, dynamic therapy was independently associated with improved survival (hazard ratio, 0.73; 95% CI, 0.53 to 0.99; *P* = .04). On inverse probability weighted analysis of 320 matched patients, the average treatment effect of dynamic therapy was to increase overall survival by 11.1 months (95% CI, 1.5 to 20.7 months; *P* = .02).

**Conclusions and Relevance:**

In this cohort study that sought to evaluate the role of adaptive dynamic therapy in localized pancreatic cancer, selecting a chemotherapeutic regimen based on response to preoperative therapy was associated with improved survival. These findings support an individualized and in vivo assessment of response to perioperative therapy in pancreatic cancer.

## Key Points

**Question:**

Is dynamic perioperative therapy associated with improved survival among patients with pancreatic cancer?

**Findings:**

In this cohort study of 322 patients with localized pancreatic cancer treated with neoadjuvant chemotherapy, selecting a chemotherapeutic regimen dictated by carbohydrate antigen 19-9 levels and/or histopathologic response was associated with significant improvement in overall survival.

**Meaning:**

This study highlights the importance of an individualized perioperative therapy for pancreatic cancer in which the choice of chemotherapy regimen is dictated by an in vivo assessment of response to neoadjuvant therapy.

## Teaser

This cohort study evaluates the association of dynamic therapy with oncologic outcomes among patients with surgically resected pancreatic cancer.

## Introduction

Pancreatic ductal adenocarcinoma (PDAC) remains a lethal disease with a rising incidence; an estimated 57 000 new cases and 47 050 deaths were recorded in the United States in 2020.^1,2^ While resection offers the best chance of cure, only 20% of patients are operable at diagnosis, most of whom will experience recurrence within 2 years.^3^ Although adjuvant therapy (AT) is considered the standard of care for operable PDAC,^4,5,6,7,8,9^ the systemic nature of PDAC has resulted in increased use of neoadjuvant therapy (NAT).^10,11,12,13^ In the latest National Comprehensive Cancer Network (NCCN) guidelines, preoperative therapy is now considered the standard of care for borderline-resectable and locally advanced disease and is an option for resectable disease.^14^

Despite its integration into modern day management of PDAC, evidence to support the superiority of an NAT approach over up-front surgery followed by AT remains lacking. Current NAT regimens, including fluorouracil, leucovorin, irinotecan, and oxaliplatin (FOLFIRINOX) and gemcitabine/nab-paclitaxel (GNp), have been extrapolated from the metastatic setting.^15,16,17^ Both regimens are associated with a near 30% response rate in metastatic disease, which, although an improvement compared with gemcitabine monotherapy (response rate, 8%-12%), implies that most tumors may not be chemosensitive. In the recent SWOG S1505 phase 2 trial, which compared perioperative FOLFIRINOX vs GNp, no improvement in overall survival (OS) with either regimen was demonstrated compared with historical data from adjuvant trials.^18^ Furthermore, recent evidence suggests that there might be 2 distinct phenotypes of pancreatic cancer (basal-like and classic epithelial subtypes) that may dictate preferential sensitivity to 5-fluorouracul (5-FU) or gemcitabine-based chemotherapy, respectively.^19,20^

Based on this, a biomarker response to therapy may prove helpful in selecting an effective chemotherapy regimen for PDAC. Recently, carbohydrate antigen 19-9 (CA19-9) level has emerged as a reliable surrogate marker for NAT efficacy in operable and metastatic PDAC with various cutoff levels correlating with survival.^21,22,23,24,25,26,27^ Indeed, evidence indicates that switching regimens in the neoadjuvant and metastatic setting to achieve CA19-9 reduction is associated with improved survival.^27,28^ Similarly, the histopathologic tumoral response (ie, pathologic response [PR]) has to NAT is also associated with survival.^29,30,31^ In combination, CA19-9 response during NAT coupled with PR in the resected specimen may provide in vivo assessment of NAT efficacy and guide AT selection, a concept herein defined as adaptive dynamic perioperative therapy (ADT).

In this analysis, we sought to establish whether ADT (achieving CA19-9 reduction during NAT and basing AT on CA19-9 response and/or histopathologic response) is associated with improved outcomes among patients with operable PDAC. With an increasing number of systemic regimens available in the neoadjuvant and adjuvant setting, we hypothesized that ADT would be associated with improved survival compared with a non-ADT approach, defined as NAT and AT regimen selection independent of CA19-9 response or PR.

## Methods

### Patient Selection

**Figure 1.**
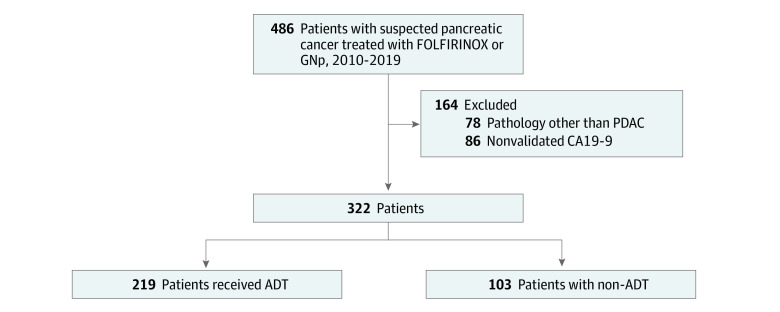
Study Flowchart ADT indicates adaptive dynamic therapy; CA19-9, carbohydrate antigen 19-9; FOLFIRINOX, fluorouracil, leucovorin, irinotecan, and oxaliplatin; GNp, gemcitabine and nab-paclitaxel; and PDAC, pancreatic ductal adenocarcinoma.

Following institutional review board approval at the University of Pittsburgh, patients with PDAC who were diagnosed between September 2010 and June 2019 and underwent curative-intent surgical resection at our institution after NAT were identified. Informed consent was exempted by the institutional review board because this is a retrospective review of a prospectively maintained database and all patient information was deidentified. Only patients who received neoadjuvant GNp or FOLFIRINOX and had complete CA19-9 data (before and after NAT) were included. In this analysis, 1 cycle of gemcitabine-based therapy was defined as either a doublet (2 weekly infusions followed by a 1- to 2-week break) or a triplet (3 weekly infusions followed by a 1-week break), while 1 cycle of FOLFIRINOX consisted of 2 treatments, 2 weeks apart. As depicted in Figure 1, patients with postresection pathology other than PDAC, those who did not have a documented baseline CA19-9 level with a concomitant reference-range level of total bilirubin (<2 mg/dL [to convert to micromoles per liter, multiply by 17.104]) at the time of diagnosis, and those with incomplete CA19-9 level following NAT (before surgery) were excluded. This study followed the Strengthening the Reporting of Observational Studies in Epidemiology (STROBE) reporting guidelines for cohort studies.

### Data Collection

Demographic, clinicopathologic, and survival data were retrieved from a prospectively maintained database. CA19-9 levels before initiation and following completion of NAT were abstracted. A CA19-9 response was defined as a 50% or greater decrease in CA19-19 level following completion of NAT based on previous evidence.^26^ At our institution, patients receiving NAT are typically restaged every 2 to 3 months with serial CA19-9 levels and pancreatic-protocol multiphasic computed tomography (CT) scans.

Pathologic stage and margin status were recorded according to the eighth edition of the American Joint Committee on Cancer (AJCC) staging manual. Margins of 1 mm or less were classified as positive (R1).^32^ PR to NAT was classified according to the College of American Pathologists (CAP) as none or poor response (CAP 3), mild to moderate partial response (CAP 2), or near-complete or complete response (CAP 1/0).^33^ A CAP score of 0/1 (complete or near-complete response) was used to define PR in this cohort.^34^

Follow-up was calculated from the date of diagnosis. Following disease recurrence, the type and duration of salvage therapy were recorded. OS was calculated from the date of diagnosis until progression, death, or last recorded follow-up.

### ADT or Non-ADT Groups

The ADT cohort included patients who either remained on or switched to an alternative neoadjuvant chemotherapy regimen guided by CA19-9 response. It also included patients for whom the AT regimen (for gemcitabine-based, gemcitabine monotherapy or GNp; for 5-FU based, either FOLFIRINOX or fluorouracil, leucovorin, and oxaliplatin [FOLFOX]; for both, gemcitabine with capecitabine) was selected based on the neoadjuvant CA19-9 response and/or PR. The non-ADT cohort were those who did not switch NAT regimen based on lack of CA19-9 response or had their AT regimen selected arbitrarily, ie, the choice of gemcitabine-based or 5-FU–based therapy in the adjuvant setting was not founded on an assessment of response (CA19-9 and/or PR). The eTable in the Supplement outlines 34 different scenarios of perioperative therapy identified in this cohort. These scenarios were ultimately grouped into 8 main groups, which were used to stratify patients into the ADT and non-ADT cohorts.

There were 4 main groups in the ADT cohort. First, patients who started on GNp and remained on gemcitabine-based therapy according to supportive evidence of response (CA19-9 level decreased by ≥50% and/or CAP 0/1 pathologic response) were included. A total of 100 patients were in this group. Second, patients who started on GNp and switched to 5-FU–based therapy because of the lack of response to the former regimen were included in the ADT group. There were 31 patients in this group. Third, patients who started on FOLFIRINOX and remained on 5-FU–based therapy according to supportive evidence of response were included in the ADT group. There were 41 patients in this group. Fourth, patients who started on FOLFIRINOX and switched to gemcitabine-based therapy owing to the lack of response to the former regimen were included in the ADT group. This group had 47 patients.

There were 4 main groups in the non-ADT cohort. First, patients who started on GNp and remained on gemcitabine-based therapy despite the lack of supportive evidence of adequate response (ie, patient had <50% decrease in CA19-9 levels and/or a CAP 2 or 3 histopathologic response) were included. There were 41 patients in this group. Second, patients who started on GNp and switched to 5-FU–based therapy despite achieving response to the initial regimen were included in the non-ADT group. There were 14 patients in this group. Third, patients who started on FOLFIRINOX and remained on 5-FU–based therapy despite the lack of supportive evidence of adequate response were included in the non-ADT group. There were 30 patients in this group. Fourth, patients who started on FOLFIRINOX and switched to gemcitabine-based therapy despite achieving response to the former regimen were included in the non-ADT group. There were 18 patients in this group. Further stratification of other groups into the ADT and non-ADT groups is outlined in the eAppendix in the Supplement.

### Statistical Analysis

Continuous variables were summarized as means with SDs or medians with IQRs. Differences between groups were tested by the 2-tailed *t* test or the Wilcoxon rank sum test, depending on the data’s distribution. When more than 2 groups were tested, 1-way analysis of variance or the Kruskal-Wallis test were used accordingly. Dichotomous or group data were summarized using raw numbers with corresponding percentages. Differences between groups were tested by χ^2^ or Fisher exact test per the data’s distribution. Survival curves were plotted using the Kaplan-Meier method, and the survival distributions were compared using the log-rank test. Multivariate logistic regression was then used to identify factors independently associated with OS. Variables with a *P* < .20 on univariate survival analysis were selected for multivariate analysis based on the stepwise backward method.

Propensity-type analysis was performed using inverse probability weights (IPWs) to assess the associations of ADT with OS owing to the potential limitations imposed by this study’s small sample size and selection bias. The weights generated by IPW were used to create weighted averages of outcomes (potential outcome means [POMs]) for the 2 study groups.^35,36,37^ The difference between the POMs of both groups was then used to estimate the average treatment effect (ATE) of ADT. Two patients were excluded from adjusted Cox regression and IPW analyses due to missing data pertaining to variables included in these models. The exclusion of those 2 patients was assumed to be at random.

All tests were 2-sided, and *P* < .05 designated statistical significance. Statistical analyses were performed using Stata statistical software version 16.0 (StataCorp).

## Results

### Demographic, Disease-Related, and Treatment Data

A total of 322 patients (mean [SD] age, 65.1 [9] years; 162 [50%] women) met inclusion criteria. The median (IQR) duration of follow-up was 49.0 (43.7-53.0) months. As outlined in the Methods section, 219 patients (68%) were allocated to the ADT group, and 103 (32%) were allocated to the non-ADT group. Baseline demographic and preoperative variables are summarized in Table 1. There were no significant differences in demographic characteristics between the 2 groups. However, the ADT cohort, compared with the non-ADT cohort, had larger mean (SD) tumor size at the time of diagnosis on CT scan and endoscopic ultrasonography evaluation (CT: 3.2 [1] cm vs 2.8 [1] cm; *P* = .005; ultrasonography: 3.0 [1] cm vs 2.7 [1] cm; *P* = .02) and higher median (IQR) baseline CA19-9 values (216 [56-800] U/mL vs 46 [16-231] U/mL; *P* < .001).

**Table 1.** Baseline Demographic and Preoperative Variables

Variable	Patients, No. (%)			*P* value
	All (N = 322)	Receiving non-ADT (n = 103)	Receiving ADT (n = 219)	
Age, mean (SD), y	65.1 (9.3)	64.5 (9.8)	66.0 (9.1)	.20
Sex				
Female	162 (50)	46 (45)	116 (53)	.16
Male	160 (50)	57 (55)	103 (47)	
BMI, mean (SD)	27.2 (5.2)	27.3 (4.9)	27.1 (5.4)	.81
ASA class				
1	1 (0.3)	1 (1.0)	0	.29
2	27 (8.4)	10 (9.7)	17 (7.8)	
3	278 (86.3)	89 (86.4)	189 (86.3)	
4	16 (5.0)	3 (2.9)	13 (5.9)	
CCI, age-adjusted, mean (SD)	4.6 (1.5)	4.6 (1.5)	4.7 (1.4)	.83
Radiologic stage at diagnosis				
Resectable	148 (46.0)	46 (44.6)	102 (46.6)	.23
Borderline resectable	159 (49.4)	53 (51.5)	106 (48.4)	
Locally advanced	15 (4.7)	4 (3.9)	11 (5.0)	
Size, mean (SD), cm				
CT	3.1 (1.2)	2.8 (1.1)	3.2 (1.2)	.005
EUS	2.9 (0.9)	2.7 (0.8)	3.0 (1.0)	.02
Type of NAC				
GNp	186 (57.8)	55 (53.4)	131 (59.8)	.28
FOLFIRINOX	136 (42.2)	48 (46.6)	88 (40.2)	
Crossover in NAC	36 (11.2)	5 (4.9)	31 (14.2)	<.001
Reason for crossover				
Lack of response	25 (69.4)	5 (100)	20 (64.5)	.52
Toxic effects	11 (31)	0	11 (35.5)	
Neoadjuvant therapy				
Cycles, median (IQR), No.	3 (2 to 4)	3 (2 to 4)	3 (2 to 4)	.19
Radiotherapy	35 (10.9)	11 (10.7)	24 (11.0)	.94
CA19-9 level, median (IQR), U/ml				
NAC				
Before	183 (48 to 681)	46 (16 to 231)	216 (56 to 800)	<.001
After	38 (16 to 92)	31 (12 to 67)	37 (15 to 103)	.03
% Change	−77 (−90 to −45)	−45 (−81 to −13)	−80 (−92 to −56)	<.001
<37 U/mL, No. (%)	137 (49.6)	43 (55.1)	94 (47.5)	.25
≥50% Reduction, No. (%)	199 (72.1)	38 (48.7)	161 (81.3)	<.001

Abbreviations: ADT, adaptive dynamic therapy; ASA, American Society of Anesthesiology; BMI, body mass index (calculated as weight in kilograms divided by height in meters squared); CA19-9, carbohydrate antigen 19-9; CCI, Charlson Comorbidity index; CT, computed tomography; EUS, endoscopic ultrasonography; FOLFIRINOX, fluorouracil, leucovorin, irinotecan, and oxaliplatin; GNp, gemcitabine/nab-paclitaxel; NAC, neoadjuvant chemotherapy.

The predominant neoadjuvant regimen used was GNp for 186 patients (58%), while FOLFIRINOX was used for 136 patients (42%), with comparable rates of either regimen and number of cycles in the ADT and non-ADT cohorts. Additionally, there was no difference in the rate of neoadjuvant radiation therapy in the ADT vs non-ADT groups (24 [11.0%] vs 11 [10.7%]; *P* = .94) (Table 1). Patients in the ADT group had a greater reduction in CA19-9 level than those in the non-ADT group (−80% [−92% to −56%] vs −45% [−81% to −13%]; *P* < .001), and 161 patients in the ADT group (81%) achieved a 50% or greater decrease in the CA19-9 level compared with 38 patients in the non-ADT group (49%; *P* < .001).

### Operative and Pathological Outcomes

As outlined in Table 2, there were no significant differences in operative data between both groups, including use of minimally invasive resection, type of procedure, and need for vascular resection. On final pathologic analysis, the ADT cohort had lower incidence of lymph node metastasis than the non-ADT cohort, but the difference was not statistically significant (131 [60%] vs 73 [71%]; *P* = .06). The ADT cohort had a lower median (IQR) number of positive lymph nodes than the non-ADT cohort (1 [0-4] vs 2 [0-4]; *P* = .046). The ADT cohort, compared with the non-ADT cohort, also had lower rates of perineural invasion (172 [79%] vs 95 [92%]; *P* = .02) and a significantly higher incidence of complete or near-complete PR (CAP 0/1; 25 [12%] vs 2 [2%]; *P* = .007). There were no significant differences in margin status, lymphovascular invasion, AJCC stage, rate of major postoperative complications, receipt of AT, number of AT cycles, total chemotherapy cycles (ie, NAT and AT), or receipt of salvage therapy between groups (Table 2).

**Table 2.** Clinicopathological and Outcome Variables

Variable	Patients, No. (%)			*P* value
	All (N = 322)	Receiving non-ADT (n = 103)	Receiving ADT (n = 219)	
Surgical approach				
Open	136 (42.2)	44 (42.7)	92 (42.0)	.87
Robotic	181 (56.2)	57 (55.3)	124 (56.6)	
Surgical procedure				
Pancreaticoduodenectomy	240 (74.5)	82 (79.6)	158 (72.2)	.26
Distal pancreatectomy	50 (15.5)	15 (14.6)	35 (16.0)	
DP-CAR	26 (8.1)	6 (5.8)	20 (9.1)	
Vascular resection				
Venous	105 (32.6)	32 (30.2)	73 (33.8)	.68
Arterial	15 (4.7)	6 (5.7)	9 (4.2)	
Both	19 (5.9)	8 (7.6)	11 (5.1)	
Pathologic tumor size, mean (SD), cm	2.6 (1.3)	2.8 (1.2)	2.6 (1.3)	.22
LN metastasis	204 (63.4)	73 (70.9)	131 (59.8)	.06
Positive LN, median (IQR), No.	1 (0-4)	2 (0-4)	1 (0-4)	.046
Total LN, median (IQR), No.	32 (24-39)	30 (23-41)	34 (24-39)	.41
LN ratio, median (IQR)	0.05 (0-0.12)	0.06 (0-0.15)	0.04 (0-0.11)	.04
Grade of differentiation				
Well	6 (1.9)	2 (1.9)	4 (1.9)	.35
Moderately	247 (78.2)	75 (72.8)	172 (80.8)	
Poorly/undifferentiated	63 (19.9)	26 (25.2)	37 (17.4)	
Lymphovascular invasion	218 (69.4)	74 (76.3)	144 (66.4)	.08
Perineural invasion	267 (82.9)	95 (92.2)	172 (78.50)	.002
Positive margins	156 (48.5)	53 (51.5)	103 (47.0)	.46
ypAJCC stage				
0	7 (2.2)	0	7 (3.2)	.21
IA/IB	100 (31.1)	29 (28.1)	71 (32.4)	
IIA	10 (3.1)	2 (1.9)	8 (3.7)	
IIB	118 (36.7)	40 (38.8)	78 (35.6)	
III	87 (27.0)	32 (31.1)	55 (25.1)	
Pathologic tumor response				
CAP 3	89 (28.1)	32 (32.1)	57 (26.6)	.007
CAP 2	201 (63.4)	69 (67.0)	132 (61.7)	
CAP 0/1	27 (8.5)	2 (1.9)	25 (11.7)	
Length of stay, median (IQR), d	7 (5-9)	7 (5-9)	7 (5-10)	.70
Mortality				
30-d	3 (0.9)	1 (0.8)	2 (0.9)	>.99
90-d	4 (1.2)	1 (0.8)	3 (1.4)	>.99
Clavien-Dindo score ≥3	67 (20.8)	21 (20.4)	46 (21.0)	.90
Adjuvant chemotherapy	227 (71.0)	72 (70.6)	155 (71.1)	.93
Chemotherapy regimen				
Gemcitabine-based	124 (39.4)	40 (40.0)	84 (39.1)	.006
5-FU–based	61 (19.4)	27 (27.0)	34 (15.8)	
Both	41 (13.0)	5 (4.9)	36 (16.7)	
Cycles, median (IQR), No.				
Adjuvant	3 (0-5)	3 (0-5)	3 (0-5)	.65
Total	6 (4-8)	6 (4-7.5)	6 (4.5-8)	.08
Adjuvant radiation	65 (20.4)	26 (25.2)	39 (18.1)	.14
Salvage therapy	159 (50.2)	50 (49.5)	109 (50.5)	.87
Follow-up duration, mean (SD), mo	49.0 (43.7-53.0)	47.1 (43.8-54.0)	49.0 (41.3-60.1)	.11

Abbreviations: 5-FU, 5-fluorouracul; ADT, adaptive dynamic therapy; CAP, College of American Pathologists; DP-CAR, distal pancreatectomy–celiac axis resection; LN, lymph node; ypAJCC, postneoadjuvant pathologic American Joint Committee on Cancer stage.

### Association of ADT With Survival

**Figure 2.**
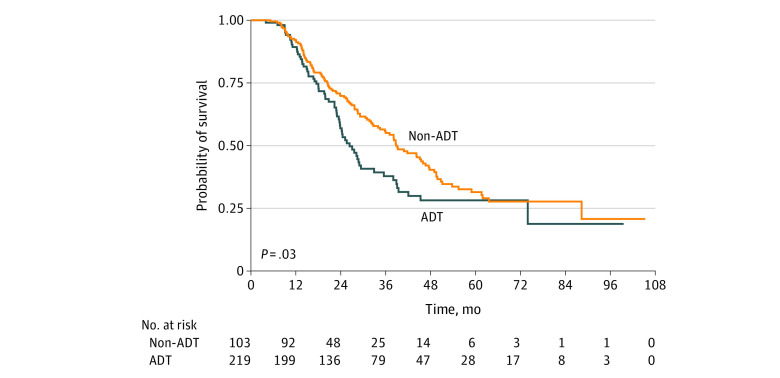
Overall Survival for 219 Patients Receiving Adaptive Dynamic Therapy (ADT) and 103 Patients Receiving Non-ADT

On Kaplan-Meier survival analysis, the median OS of the ADT group was significantly higher than that of the non-ADT group (38.7 months [95% CI, 34.0-46.7 months] vs 26.5 months [95% CI, 23.5-32.9 months]; *P* = .03) (Figure 2). On multivariate analysis (Table 3), ADT was found to be associated with a 27% reduction in the hazard of death (hazard ratio [HR], 0.73; 95% CI, 0.53-0.99; *P* = .04). In this model, AT receipt was also associated with a reduced hazard of death (HR, 0.53; 95% CI, 0.39-0.73; *P* < .001), while borderline-resectable disease (HR, 1.44; 95% CI, 1.00-2.06; *P* = .047) and R1 margins (HR, 1.49; 95% CI, 1.10-2.03; *P* = .01) were associated with an increased risk of death.

**Table 3.** Factors Associated With Overall Survival

Factor	HR (95% CI)	*P* value
Adaptive dynamic therapy	0.73 (0.53-0.99)	.04
Age	1.01 (1.00-1.03)	.11
Resectability		
Resectable	1 [Reference]	NA
Borderline resectable	1.44 (1.00-2.06)	.047
Locally advanced	1.01 (0.59-1.73)	.97
Vascular resection		
None	1 [Reference]	NA
Arterial	1.11 (0.94-1.30)	.21
R1 margin	1.49 (1.10-2.03)	.01
Adjuvant chemotherapy	0.53 (0.39-0.73)	<.001

Abbreviations: HR, hazard ratio; NA, not applicable.

IPW estimators were used to examine the ATE of ADT on OS. Following matching of preoperative, pathologic, and treatment variables (age, body mass index, vascular resection, pathology, margin status, and AT receipt) in 320 patients, the ATE of ADT was to increase OS by 11.1 months (95% CI, 1.5-20.7 months; *P* = .02) vs the ATE of 25.0 months (95% CI, 20.0-30.1 months) in the non-ADT group.

## Discussion

This study suggests that an adaptive approach to multimodal treatment of operable pancreatic cancer is associated with improved survival. ADT involves achieving CA19-9 response during NAT (defined as a 50% decrease in CA19-9 levels from baseline) either by using an effective first-line neoadjuvant regimen (GNp or FOLFIRINOX) or by switching regimens in the absence of CA19-9 response (from GNp to FOLFIRINOX or vice versa) and tailoring AT according to the CA19-9 response and/or PR to NAT. Our analysis suggests ADT is associated with regional lymph node sterilization, reductions in perineural invasion and lymphovascular invasion, higher rates of complete or near-complete PR, and improved overall survival. These findings indicate that even in the era of effective chemotherapy, a one-size-fits-all approach is unlikely to improve survival for operable PDAC, unless treatment is based on an in vivo assessment of response.

NAT is now an acceptable option for the treatment of operable PDAC. Despite its purported benefits, it has not demonstrated a clear survival benefit over a surgery first, followed by AT approach. The PREOPANC trial randomized patients with borderline resectable PDAC to neoadjuvant chemoradiation vs up-front surgery followed by AT and showed no survival advantage for NAT.^38^ Survival was discouraging for both arms (16.0 vs 14.3 months; *P* = .01) and was partly attributed to use of single-agent gemcitabine; however, an updated longitudinal analysis of this trial revealed an improvement in the 5-year OS in favor of neoadjuvant chemoradiotherapy (20.5% vs 6.5%; HR: 0.73; *P* = .03).^39^ Instigated by 2 randomized clinical trials in the metastatic setting demonstrating a near doubling of survival using multidrug regimens such as FOLFIRINOX and GNp^15,16,17^ vs gemcitabine alone, the phase 2 SWOG 1505 study randomized patients with operable PDAC to perioperative FOLFIRINOX or GNp.^18^ Although this trial showed comparable efficacy of both regimens, median survival on an intention-to-treat basis was modest and remained comparable with historical adjuvant therapy trials.

A potential reason for these modest results was the lack of in vivo assessment of response to NAT. Although FOLFIRINOX and GNp exhibit improved response rates (approximately 30%) compared with gemcitabine monotherapy (8%-12%), most patients are unlikely to be responders. It is anticipated that several ongoing trials (NEPAFOX,^40^ NEONAX,^41^ NorPACT-1,^42^ PANACHE01-PRODIGE48,^43^ ESPAC-5F^44^) and the upcoming ALLIANCE A021806 clinical trial will shed further light on the efficacy of various regimens. However, in the absence of an adaptive in vivo assessment of response and recent translational evidence suggesting 2 distinct phenotypes of pancreatic cancer (basal and epithelial) that preferentially respond to 5-FU– or gemcitabine-based regimens, respectively,^19,20,45^ it is possible that these trials will not demonstrate major improvements in survival given that none have a built-in option of switching chemotherapy regimen based on tumor chemosensitivity.

Assessment of chemotherapy response in PDAC can be challenging, but serum CA19-9 is useful biomarker of response during neoadjuvant and definitive therapy.^21,22,23,24,25,26,27^ Tzeng et al^25^ evaluated 141 patients with pre- and post-NAT CA19-9 levels and found that decrease of CA19-9 to the reference range (<40 U/ml) was associated with improved OS in both the nonresected and resected cohorts. Failure of response (ie, CA19-9 remained stable or increased) was a predictor of unresectability and development of metastatic disease.^25^ Truty and colleagues^28^ evaluated predictors of survival in patients with borderline-resectable and locally advanced PDAC who were treated with total NAT. Of the 194 patients analyzed, 36 (19%) required a switch in neoadjuvant regimen primarily because of lack of adequate response. Improved survival was associated with decrease of CA19-9 levels to the reference range and achieving a metabolic response on positron emission tomography. On multivariate analysis, survival was associated with prolonged duration of neoadjuvant chemotherapy (≥6 cycles), post-NAT CA19-9 decrease to reference range, and near-complete or complete PR. We also previously identified the important role of CA19-9 response during NAT.^24,26^ In our experience, a 50% or greater decrease in CA19-9 level was associated with improved survival, while a reduction of 50% or greater and decrease of CA19-9 levels to the reference range during NAT was associated with optimal survival.^24,26^ Based on these findings, our group and others have proposed that a lack in CA19-9 response should prompt a chemotherapy switch during NAT (FOLFIRINOX to GNp, or vice versa). Although the optimal magnitude of CA19-9 response remains to be identified, a 50% cutoff—based on the previously described data—is often used as a surrogate for in vivo chemosensitivity and was the cutoff chosen in this analysis.

Although CA19-9 level change is an effective surrogate marker of response, as many as 18% to 34% of patients with PDAC have Lewis α-β- (negative) blood genotype and present with a normal level of CA 19-9 (<37 U/ml).^46^ In the absence of a dependable biomarker, histopathologic grading of the extent of residual cancer may be used as a surrogate marker of response to NAT.^29,30,31,34,47,48^ Chatterjee et al^34^ concluded that the grade and extent of residual tumor is critical in predicting prognosis in PDAC treated with NAT. Although several scoring systems for pathologic response have been proposed, the authors used the CAP and Evans score to demonstrate a significant survival advantage for patients with complete or near-complete response (CAP 0/1 or Evans grade III/IV) vs partial or minimal response (CAP 2/3 and Evans I-IIb).^34^ Based on this data and the heterogeneity of grading CAP 2 tumoral response, complete or near-complete response (CAP 0/1) was used to define PR in this study.

The findings presented in this analysis are congruent with a similar study performed by Alva-Ruiz and colleagues.^49^ The authors performed an analysis of 468 patients with operable PDAC treated with neoadjuvant chemotherapy, of whom 30% underwent a switch in regimen. They noted no difference in OS between patients resected after first-line treatment and those who underwent a chemotherapy switch. Notably, on adjusted analysis, CA19-9, PR, and AT receipt were some of the factors associated with OS, although the type of AT regimen was not a parameter evaluated in the study. Our analysis further expanded on this concept and evaluated patients who either remained on or switched to an alternative neoadjuvant regimen dictated by CA19-9 response and those who remained or switched to an alternative adjuvant regimen based on both the CA19-9 response and/or PR (ADT group). This group had a significant improvement in survival, as supported by multivariate analysis and IPW estimators. Taken together, both analyses support the emerging concept that a dynamic assessment of response during and following NAT may improve survival.

### Limitations

Several limitations of this study should be acknowledged, most important of which is that the analysis was restricted to a select group of patients who underwent resection following NAT and does not represent the intention-to-treat cohort. Data on patients who started NAT but failed to reach resection due to disease progression or decline in performance status were excluded because they could not have undergone AT. This likely explains why survival in both ADT and non-ADT groups was higher than intention-to-treat cohorts previously reported in the literature (such as the SWOG S1505 study^18^). Also, because of the retrospective study design, we could not account for treatment decisions, including which regimen was initiated, when chemotherapy was switched, use of radiation, timing of surgery, and the decision to treat with AT. These limitations can only be addressed in the context of a clinical trial.

## Conclusions

To our knowledge, this is the first analysis to examine the association of a personalized adaptive treatment strategy for localized PDAC using in vivo assessment of response to NAT with OS. The current analysis supports the selection of both NAT and AT regimen type based on response to contemporary NAT. Although prospective validation is warranted, the findings suggest that an adaptive approach to systemic treatment for operable pancreatic cancer improves survival.

## Notes

Supplement. **eAppendix.** Special Considerations **eTable.** All Potential Scenarios of Perioperative Therapy in the Cohort
