# Errors in Figure 2

**DOI:** 10.1001/jamanetworkopen.2022.27318

**Published:** 2022-07-25

In the Original Investigation titled “Adaptive Dynamic Therapy and Survivorship for Operable Pancreatic Cancer,”^1^ published June 23, 2022, the lines in Figure 2 were mislabeled. The orange line should have been labeled ADT, and the blue line should have been labeled non-ADT. This article has been corrected.^1^
