# Supplementary material for: Adaptive Dynamic Therapy and Survivorship for Operable Pancreatic Cancer
Source: JAMA Netw Open. 2022 Jun 23;5(6):e2218355. doi: 10.1001/jamanetworkopen.2022.18355 (PMC9227002; doi:10.1001/jamanetworkopen.2022.18355)
Supplement: Supplement. — eAppendix. Special Considerations eTable. All Potential Scenarios of Perioperative Therapy in the Cohort [file jamanetwopen-e2218355-s001.pdf]

Supplemental Online Content

AlMasri S, Zenati M, Hammad A, et al. Adaptive dynamic therapy and survivorship for operable pancreatic cancer. *JAMA Netw Open.* 2022;5(6):e2218355. doi:10.1001/jamanetworkopen.2022.18355

eAppendix. Special Considerations

eTable. All Potential Scenarios of Perioperative Therapy in the Cohort

This supplemental material has been provided by the authors to give readers additional information about their work.

© 2022 AlMasri S et al. *JAMA Network Open.*

eAppendix. Special Considerations

1) Seventy-nine patients were CA19-9 non-secretors at baseline (CA19-9<37U/ml). Allocation to dynamic and non-dynamic therapy in this cohort was based on pathologic response. For instance, a patient who received gemcitabine/nab-paclitaxel in the neoadjuvant setting, had a CAP 0/I pathologic response and received gemcitabine-based therapy in the adjuvant setting, this was considered dynamic therapy. If this patient had a CAP II or III pathologic response and switched to 5-FU based therapy in the adjuvant setting, this was also considered dynamic therapy. However, if the patient had a CAP II or III on GA and remained on gemcitabine-based therapy adjuvantly, this was considered non-dynamic therapy.
2) Thirty-six patients had crossover in their neoadjuvant regimen (gemcitabine/nab-paclitaxel to FOLFIRINOX and vice versa). The two reasons for this switch were stratified into either lack of response (25 (69%))-either on radiologic assessment and/or CA19-9- or toxicity to the first regimen (11 (31%)) as outlined in **Table 1**. For both groups, allocation to the non-dynamic and dynamic therapy groups was based on the CA19-9 response prior to this switch; if the patient had a ≥50% drop in their CA19-9 and switched, this was considered non-dynamic therapy as the switch happened despite a supportive evidence of response to the first regimen.
3) For patients who did not receive adjuvant therapy (n=93), allocation into dynamic vs non-dynamic therapy also factored in the reasons for not receiving adjuvant therapy. If the patient completed his therapy preoperatively or declined adjuvant therapy but yet did have adequate CA19-9 and/or pathologic response, this was considered dynamic therapy

© 2022 AlMasri S et al. *JAMA Network Open.*

even though the patient might not have completed a sufficient duration of treatment. If the patient declined adjuvant therapy and didn't have an adequate response to neoadjuvant therapy, this was classified as non-dynamic therapy. For patients who experienced rapid disease progression following surgical resection or didn't receive adjuvant therapy due to decline in performance status, allocation into the two study groups was based on whether the patient did or did not achieve an adequate tumor response to neoadjuvant therapy (supplemental table 1)

4) CA19-9 was used as a primary determiant for allocation into the two groups. For instance, a patient who had CA19-9 response to one regimen but had a suboptimal PR (CAP III/II) and remained on the same adjuvant regimen, this was considered adaptive therapy. If a patient did not have a CA19-9 response but a PR (CAP 0/I) to the neoadjuvant regimen and remained on the same regimen in the adjuvant setting, this was considered nonadaptive therapy.

eTable. All Potential Scenarios of Perioperative Therapy in the Cohort

Scenario	NAT regimen (0=GNp, 1=FFX)	NAT crossover 0=no, 1= yes	CA19-9 ≥50% and/or near/complete PR	AT: none=0 Gem-based=1, 5FU based=2, both=3	ADT
1	0	0	1	1	1
2	0	0	1	3	1
3	0	0	1	0	1
4	0	0	0	0	0
5	0	0	0	1	0
6	0	0	0	2	1
7	0	0	0	0	0
8	0	0	0	1	0
9	0	0	0	2	1
10	0	0	0	1	0
11	0	0	0	3	1
12	0	1	0	0	0
13	0	1	0	3	1
14	0	0	1	2	0
15	0	1	1	3	1
16	1	0	1	0	1
17	1	0	1	3	1
18	1	0	0	0	0
19	1	0	0	0	0
20	1	0	0	1	1
21	1	0	0	1	1
22	1	0	1	2	1
23	1	0	0	2	0
24	1	0	0	NA	0
25	1	0	0	0	0
26	1	0	0	1	1
27	1	0	0	2	0
28	1	1	1	0	1
29	1	1	1	3	0
30	1	1	1	1	1
31	1	1	NA	1	0
32	1	1	1	1	1
33	1	1	0	0	1
34	1	1	1	1	0

NAT: neoadjuvant therapy, GNp: gemcitabine/nab-paclitaxel, FFX: FOLFIRINOX, PR: pathologic response, AT: adjuvant therapy, ADT: adaptive therapy.

© 2022 AlMasri S et al. *JAMA Network Open.*
